# Reviews

**Published:** 1880-08

**Authors:** 


### Reviews.

**Modern Medical Therapeutics; a Compendium of Recent Formulæ and Specific Therapeutical Directions from the Practice of Eminent Contemporary Physicians, American and Foreign.** By Geo. H. Napheys, A. M., M. D., &c. Seventh Edition, Enlarged and Revised. Philadelphia: D. S. Brinton, 115 South Seventh St. 1880.

This work is well known to nearly every physician. The fact of its having reached the seventh edition is a stronger recommendation than any we could possibly write. That works of this character are many times found to be extremely useful, is undoubtedly true. Whatever may be one's views regarding the thoroughly empirical practitioner,—one who has a large number of set prescriptions with which to battle disease—it cannot be denied that there are few physicians who have not one or more pet formula with which they would be loath to part. This being admitted, it follows that to have easy access to the favorite formulas of eminent medical men, must be considered as a positive advantage. V. P.

---

**A Guide to the Practical Examination of Urine, for the use of Physicians and Students.** By JAMES TYSON, M. D., Professor of General Pathology and Morbid Anatomy in the University of Pennsylvania. Third edition, revised and corrected, with illustrations. Philadelphia: Lindsay & Blakiston. 1880.

The practical value of this manual of Dr. Tyson is shown by the rapidity with which new editions of the work have been called for. We are glad to see that the importance of the examination of urine as a means of diagnosis, is becoming better appreciated. The technical difficulties, which for the busy physician the larger treatises rather increase than remove, are in this little book so simplified and explained as to enable the student and practitioner to reach successful results with but little practice. D.

---

**The Utricular Glands of the Uterus and the Glandular Organ of New Formation which is developed during Pregnancy in the Uterus of the Mammalia, including the Human Species.** By GIOVANNI BATTISTA ERCOLANI, Permanent Secretary of the Academy of Sciences of Bologna, &c., &c., to which is appended his monograph upon the unity of the anatomical type of the placenta in all the mammalia, and the physiological unity of the nutrition of the foetus in all the Vertebrata. also a general summary and classification, written expressely for this edition. With a quarto atlas of fifteen plates, engraved by Bettini and reproduced by the Heliotype process. Translated from the Italian under the direction of Henry O. Marcy, A. M., M. D. Boston: Houghton, Osgood & Co., The Riverside Press, Cambridge. 1880.

**Atlas of Memoir upon the Utricular Glands of the Uterus, &c. &c.** Consisting of ten plates, also a monograph upon the Unity of the anatomical type of the Placenta. Consisting of five plates. By Professor G. B. ERCOLANI, of Bologna. Engraved by Bettini and reproduced by the Heliotype process. Boston: Houghton, Osgood & Co., The Riverside Press, Cambridge. 1880.

The above comprehensive title unfolds the objects designed to be accomplished by the author in treating of these important and interesting subjects. The translator states that his task was

"undertaken from the conviction that their publication would be of service to science."

The physiology of reproduction is one of the most interesting problems offered for solution to the scientist. In attempting to unfold the unity of the anatomy of the placenta in the mammalia, and of the nutrition of the embryo in the vertebra, a field of study has been entered upon, which we think will unfold facts of rare interest.

In this brief review we can not do justice to the value of the investigations of Prof. Ercolani. The results here unfolded need careful perusal and study, and if supplemented by experimental examination in the same direction, under the guidance of the facts and principles elucidated by these patient investigations, the student cannot fail to be profited thereby. To those favored with leisure, whose tastes are directed to the channel of physiological study and research, and who, unlike the busy practitioner, seek diversion from pathological and therapeutical labors, in an allied science, there will be found here, a broad scope for study, and a profitable return for research.

The atlas is a beautiful example of the accuracy of the process, by which the glandular structure of the uterus and placenta are brought out. Every tissue is copied, the structure of important organs are illustrated, and parts hitherto "veiled in mystery" are clearly demonstrated.

The typographical execution of the works is in the best style of the Riverside Press, and we especially commend the publishers for their enterprise in affording the profession an opportunity to study the subject which it is the design of these works to elucidate.

L.

---

**The Black Arts in Medicine.** With anniversary address by JOHN D. JACKSON, A. M., M. D. Edited by L. S. MCMURTRY, A. M., M. D. Cincinnati: Robert Clarke & Co. 1880.

Dr. McMurtry has done a good work in reproducing this admirable little essay of Dr. Jackson's, and we wish that the work might be read by every physician. The only individuals who would not enjoy it might be those whose consciences might prick them on its perusal. The picture here so skillfully portrayed of the littleness, scheming and lieing to which some men descend to get a vantage ground in the race for professional success, would be a painful one, if we believed that the knaves in the profession were more than the few. The book is handsomely printed and bound, and will be sent by the publishers by mail on receipt of one dollar. D.

---

**Photographic Illustrations of Skin Diseases.** By GEORGE HENRY FOX, A. M., M. D. Clinical Professor of Dermatology, Sterling Medical College, Columbus, Ohio, &c. Forty-eight colored plates taken from life. New York: E. B. Treat, 805 Broadway. Parts 11 and 12.

Part 11 of this valuable work contains photographic illustrations of Herpes Facialis, Hydroa Bullosum, Erythema circinatum, Erythema exfoliativum, and Purpura Simplex.

Part 12 contains photographic plates, illustrating Cornua Cutanea, Alopecia Areata, Morphœa, Scleroderma and Sarcoma Pigmentosum.

Prof. Fox has fulfilled the assurances given to the profession, when he undertook the work, now successfully completed, and has contributed the most useful and practical work for the study of skin diseases yet offered to the public. The illustrations are accurate, the selections are judiciously made, and the entire work will be of greater assistance to the student or to the general practitioner than any other with which we are acquainted. We are gratified to add that the author intends to issue a companion work entitled "Photographic Illustrations of Cutaneous Syphilis," which will supplement the work which he has just completed. This addition will be a valuable contribution to the study of a class of diseases frequently met within our large centres of population. L.

